# Recent Progress in Surgery

**Published:** 1877-01

**Authors:** J. C. Warren


Recent Progress in Surgery.—*By J. C. Warren, M. D.—Subcutaneous Osteotomy.*—The recent improvements in the operation of division of the upper extremity of the femur for anchylosis of the hip-joint were set forth in a paper read by Mr. Maunder before the Clinical Society of London in May last. In the *Medical Times and Gazette* for July 22, 1876, is an account of two operations performed by Mr. Maunder by the new method, which appears to be much more easily carried out than the old one, where a fine saw was used for dividing the bone, as recommended by Mr. Adams. Two patients were submitted to this treatment; one was a young girl who for about seven years had been unable to put her foot to the ground. Disease of the hip-joint had ended in fibrous anchylosis, with the thigh fixed at an angle of one hundred and eighteen degrees with the trunk. Thomas' splint had been tried for several weeks with the view of gradually straightening the limb, but no improvement whatever had resulted. The other patient was a young man of fine proportions and well nourished, who had been sent up from Plymouth with the express object of undergoing the operation. Disease of the left hip-joint had supervened upon fever, and had ended in fibrous anchylosis with the leg at right angles with the trunk. Before commencing the operation, an assistant standing in front of the patient drew forwards the soft parts. Mr. Maunder then measured the distance from the top of the trochanter major to the shaft at a level immediately below the small trochanter—this spot being selected because it is the highest beyond the attachment of the numerous muscles which are inserted into the upper end of the femur. At this spot (and while the soft tissues are well drawn forwards) he inserts a double-edged knife down to and at right angles with the bone on the outer side of the limb, cuts through the periosteum, and then, before removing the knife, introduces the chisel, which is also kept at right angles to the axis of the shaft of the femur. With a light wooden mallet the chisel is driven well into the bone, then partially withdrawn, to be again driven onwards, inclined somewhat obliquely forwards, and then backwards, so as to divide the bone in the rest of its thickness. While doing this the hand of another assistant is pressed upwards against the inner surface of the thigh, so as to make counterforce to the direction of the penetrating chisel. Finally, the limb is gradually and carefully extended, any small portion of bone which may happen to have escaped the chisel being at the same time broken down; lastly, a straight interrupted outside splint is applied.

The chisel—a separate one for each case—used by Mr. Maunder is three-eighths of an inch in width at the cutting edge, where it is wider than elsewhere, and three inches and a half long in the shaft. The operation is attended with next to no hæmorrhage, and the small wound in the soft tissues through which the chisel has been worked becomes valvular and air-tight as soon as the tissues themselves are allowed to fall backwards into their natural position. A minute or two was the time required to complete the division of the bone in the case of the girl; in that of the man the process was longer, owing to the greater thickness and toughness of the bone.

In three cases in which the operation had been performed several weeks previously, the patients were already able to walk—one man without the aid of stick or crutch—with limbs in nearly perpendicular positions, and with little or no lordosis. There necessarily, however, remains some deformity about and around the hip-joint. This is easily understood when it is remembered that there is anchylosis at an angle, and in some cases it has followed so-called dislocation from disease; while, as the division of the femur is made below the small trochanter, there is no attempt to correct the abnormal position of the upper extremity of the bone.

Mr. Maunder stated that in most of his cases there has been no suppuration whatever after the operation, and that it was very limited indeed in the case in which it occurred. This entirely coincides with the experience of Professor Volkmann, who also has employed the chisel instead of the saw. Professor Volkmann, however, used three chisels of different thickness to prevent the jamming and sticking fast in the deeper parts of the incision into the bone. The superficial part was divided with the stoutest, the deeper with a thinner, and the deepest with the thinest instrument of all, so that the cleft was slightly wedge-shaped. Mr. Maunder, by a modification of the form of the chisel, finds it unnecessary to use more than one instrument.

The instrument used by him is called a carver's "cold" chisel. At a recent meeting of the Clinical Society Mr. Maunder reported all of the cases operated upon in this way, seven in number; six of these had been done to correct deformity after hip-joint disease, and one when the shaft of the femur had been divided about its middle to correct deformity following mal-united fracture in a man aged thirty. In two of the earlier cases slight suppuration had taken place, while in the other five the wounds healed as in tenotomy, the patients getting about from six to eight weeks after the operation.

This method has been employed by Billroth in rachitic deformities of the leg, and in club-foot. In a case recently reported this operation was performed upon both tibiæ of a young man eighteen years of age with success. All straightening gained is immediately secured and maintained by a plaster of Paris dressing. Dr. Ashurst of Philadelphia has recently divided the neck of the femur on one side of a young person after Adams' method, and on the other side, below the trochanter, after Gant's method, the patient making a rapid recovery.

*Gastrotomy.*—A successful case of gastrotomy for stricture of the œsophagus has been reported recently by M. Verneuil, of Paris. An account of this operation will be found in the *Journal*, December 7, 1876.

*Partial Resection of the Stomach.*—This operation was originally suggested by Merrem, in 1810, in a cancer of the pylorus, and the operation was practiced by him on three dogs, of which one died the next day, one at the end of twenty-two days, and one at the end of twenty-seven days.

The authors of this paper endeavored to determine by experiment, first, whether extirpation of the pylorus was necessarily fatal; and, second, whether there were cases in which cancer of the pylorus could be entirely removed by this operation without danger of return or of generalization of the disease. Gastrotomy was performed, for this purpose, on seven dogs, and in each case a circular portion of the pyloric extremity of the stomach was removed. There were two methods of doing this suggested: either to produce adhesion of the stomach to the abdominal wall and subsequently to reach the disease without opening the peritoneal cavity, or to resort to simple gastrotomy. The latter method was employed. An incision was made from the tip of the xiphoid cartilage to the umbilicus, the pylorus was drawn through the wound, and the portion removed while the divided ends of the pyloric portion and the duodenum were held firmly closed by an assistant, to prevent the escape of their contents into the abdominal cavity. The divided surfaces were then brought together with sutures. It was found that silk sutures obtained better results than catgut sutures. Pains were taken to bring the mucous as well as the serous surfaces very accurately in contact. Two of the seven dogs alone survived this operation. One died eight months after the operation; at the autopsy a deep ulceration was found at the point of the cicatrix. The other was killed at the end of five months. The cause of death in the other cases was peritonitis, produced in two cases by opening of the abdominal cavity by rupture of the sutures; in two other cases by escape of the contents of the intestine into the abdominal cavity; in one case by an improperly applied suture, and in the other by a separation of the cicatrix. In the two successful cases no stricture was formed at the point operated upon. The authors collected a large number of cases of cancer of the stomach observed in Vienna from 1817 to 1873. From these statistics it was found that the number in which there were no complications to contra-indicate the operation was an exceedingly small one.

*Exsection of the Pancreas.*—This operation was performed by Dr. Justin, of Sebastopol, California, assisted by Dr. B. B. Allen, who reports the case. The patient was an Indian, thirty-five years old, who had been stabbed in several places. The pancreas was found protruding from an orifice a little in front and above the left kidney. It had been exposed about twelve hours, and was so altered that it was thought unadvisable to replace it. It remained untouched for two days, when a ligature was put around the base of the protruding mass, and a portion seven inches in length was removed. Nineteen days later, the ligature having come away, and the remains of the organ having retracted into the abdomen, the wound closed. The patient appears to be in no way affected by the loss of the organ. The writer says: "Whilst the pancreas was dangling from the orifice it was still secreting a greasy fluid, and, in handling it, my hands became greasy." It is to be regreted that no more satisfactory proof of the identity of the mass removed with the pancreas was given by the writer.

*Operation for Cleft Palate with the Object of Obtaining a Clear Speech without the Nasal Tone.*—The author reviews the various operations for cleft palate, and points out the difficulties in each by which the nasal tone is still preserved. The insufficiency of the new palate acting as a valve to separate the nasal from the buccal cavity is the cause of the nasal tone. This insufficiency is caused by the immobility and shortness of the new palate, which prevents it coming in contact with the posterior wall of the pharynx with sufficient accuracy to exclude the passage of air. Passavant attempted to remedy this difficulty by separating the soft from the hard palate by a cross-cut, which leaves the velum freely movable; its lower edge is then refreshed and stitched to the lower wall of the pharynx. Another device of the same surgeon was to free the velum by two lateral incisions extending forwards from the base of the soft palate close to the alveolar processes, the two being united by a cross incision; the flap so formed was dissected up and set back. A communication between the mouth and nose was thus left through the bony fissure, and must be closed by a future operation. These operations have been tried in but one or two cases, and with partial success only. Dr. Mason, of London, has freed the tense velum by lateral incision from the posterior border forwards, converting the velum into a large uvula. This had the effect of relieving the tension, but the valve was still insufficient, and the nasal tone remained. Schonborn lengthened the palate by a flap taken from the posterior wall of the pharynx, and stitched to the lower border of the velum. After union the base of the flap was cut away. The author proposes an operation based on the action of the superior constrictor muscle of the pharynx, which renders this portion of the pharynx quite prominent when the palate is lifted to meet it. A permanent semi-circular prominence is effected by the cicatrization resulting from an excision of a band of mucous membrane from this portion of the pharynx. The portion removed may extend partially or completely across the pharynx. The posterior wall of the pharynx is thus brought nearer to the palate, while the contraction of the transverse cicatrix would counteract the tendency of the longitudinal cicatrix in the newly-formed palate to contract. The contraction of this latter is supposed to shorten a palate which would otherwise be sufficiently long to close the passage. It will be noticed that Schonborn, by taking his flap from the posterior wall of the pharynx, not only lengthens his palate, but produces the desired cicatricial prominence of the pharyngeal wall. The author recommends his method for cases which have already been operated upon by the old methods without improvement of the voice.—*Boston Medical and Surgical Journal.*

